# Walking and Running of Children with Decreased Femoral Torsion

**DOI:** 10.3390/children11060617

**Published:** 2024-05-22

**Authors:** Christos Tsagkaris, Marry E. Hamberg, Christina Villefort, Thomas Dreher, Britta K. Krautwurst

**Affiliations:** 1 Pediatric Orthopedic and Trauma Surgery, Children’s University Hospital Zürich, 8032 Zürich, Switzerland; marina.hamberg@physio-hin.ch (M.E.H.); ch_villefort@hotmail.com (C.V.);; 2 Pediatric Orthopedics, Balgrist University Hospital, University of Zurich, 8008 Zürich, Switzerland

**Keywords:** femoral torsion, running, children, motion analysis, kinematics, femoral retroversion, femoral retrotorsion

## Abstract

Understanding the implications of decreased femoral torsion on gait and running in children and adolescents might help orthopedic surgeons optimize treatment decisions. To date, there is limited evidence regarding the kinematic gait deviations between children with decreased femoral torsion and typically developing children, as well as the implications of the same on the adaptation of walking to running. A three-dimensional gait analysis study was undertaken to compare gait deviations during running and walking among patients with decreased femoral torsion (*n* = 15) and typically developing children (*n* = 11). Linear mixed models were utilized to establish comparisons within and between the two groups and investigate the relationship between clinical examination, spatial parameters, and the difference in hip rotation between running and walking. Patients exhibited increased external hip rotation during walking in comparison to controls, accompanied by higher peaks for the same as well as for knee valgus and external foot progression angle. A similar kinematic gait pattern was observed during running, with significant differences noted in peak knee valgus. In terms of variations from running to walking, patients internally rotated their initially externally rotated hip by 4°, whereas controls maintained the same internal hip rotation. Patients and controls displayed comparable kinematic gait deviations during running compared to walking. The passive hip range of motion, torsions, and velocity did not notably influence the variation in mean hip rotation from running to walking. This study underlines the potential of 3D gait kinematics to elucidate the functional implications of decreased FT and, hence, may contribute to clinical decision making.

## 1. Introduction

Deformities of the lower extremities are common causes of gait abnormalities and subsequent parental concerns among children and adolescents [1]. Rotational irregularities, such as decreased femoral anteversion, also known as decreased femoral torsion (FT), may result in an out-toeing gait, limitations in daily activities, such as running, and pain in the hip, knee, or feet [2]. It is worth mentioning that the terms “torsion” and “version” and their derivatives are interchangeably used in the literature and have stirred controversy regarding the precise description of the twisting of a long bone along its longitudinal axis. Although “torsion” is considered the most appropriate term to describe the twisting of a bone at a given level, derivatives of version, such as femoral ante- and retroversion, tend to be established as an accurate expression of the “total” femoral torsion [3,4].

From an epidemiologic point of view, decreased FT seems to occur more commonly in obese children and in children suffering from particular syndromic conditions or hip pathologies, including the epiphysiolysis of the femoral head [5,6,7,8]. Reduced FT refers to the twist between the proximal and distal parts of the femur on the transverse plane [9]. Normal FT depends on age and gender and evolves towards a normal mean value of 15° femoral anteversion in adulthood [1,10,11].

Patients with decreased FT have an unusual torsional profile upon clinical examination, with decreased hip internal rotation and increased external rotation [6,12,13]. Most rotational abnormalities typically improve during the first year of walking or resolve spontaneously as the child grows [14,15,16,17,18]. Emerging evidence also suggests that rotational deformities of the femur that are not addressed during adolescence are associated with an increased risk for femoroacetabular impingement (FAI) or early hip-osteoarthritis [19,20]. Biomechanically, the position of the femoral head can lead to an anatomical conflict with the acetabulum, inducing FAI [20,21,22]. Patients with decreased FT experience functional limitations in hip flexion, abduction, and internal hip rotation; additionally, hip and knee pain during gait may result in difficulties in daily activities and sport [17,19,21]

Conservative treatments have not shown the potential to modify the anatomical configuration of the femur; therefore, the correction of torsional deformities can only be achieved surgically with a rotational osteotomy. The indications as well as the time of corrective surgery remain challenging [22,23,24,25].

Currently, information regarding decreased FT derives either from clinical examination techniques or imaging modalities such as torsional magnetic resonance imaging (MRI) or computed tomography (CT). These methods are static and fail to capture the dynamic components of gait. Three-dimensional (3D) motion analysis is a well-established dynamic measurement method for describing and objectively measuring deviations in gait kinematics and kinetics, which is often used for clinical decision-making and recommended for planning surgical correction of a wide range of orthopedic conditions and rotational deformities [24,25]. Motion analysis provides clinicians and researchers with measurements of the mobility of the hip, knee, and ankle joint and thus contributes to quantifying discrete radiological findings such as hip deformities or irregular femoral torsion values to real-life walking and running capacity and limitations. In this manner, it can improve the understanding of orthopedic biomechanics, while informing clinicians about functional limitations patients experience and enhancing decision-making on this basis.

Gait deviations for increased FT have been researched [26,27], but reports on decreased FT in children are scarce. A case series involving four adults with Slipped Capital Femoral Epiphysis (SCFE) and decreased FT indicated increased FAI, but it has been reported as too small to establish statistically significant differences in the gait analysis [4,17]. To the best of our knowledge, relevant evidence about children derives from a case report of an adolescent with unilateral hip pain, whose gait analysis exhibited abnormal hip and knee flexion during the swing phase of gait [9]. Apart from regular gait, running is an activity worth investigating because it is a pattern of motion that changes the allocation of loading to the joints of the lower extremities and might particularly increase the valgus stress at the knee [28].

To address this knowledge gap, a standardized 3D motion analysis protocol is used to document the implications of decreased femoral torsion on walking and running gait among children. The purpose of this study is twofold: firstly, to examine walking and running deviations among children with decreased FT; secondarily, to juxtapose these observations with those made among TDC. We hypothesize that children with femoral retroversion exhibit gait deviations in comparison to typically developing children. We further hypothesize that femoral retroversion can have an impact on gait alterations observed between walking and running among typically developing children.

## 2. Materials and Methods

### 2.1. Participants

A total of 26 children and adolescents underwent a standardized 3D motion analysis. Children and adolescents with symptomatic decreased femoral anteversion were recruited from the pediatric orthopedic outpatient clinics in the author’s institutions and examined in a 3D Gait and Motion Analysis Laboratory. The inclusion criteria for all participants were as follows: (1) age between ≥8 and <18 years, (2) suitability for the test intervention with the ability to run; and (3) written informed consent by the adolescent and/or the legal representative. Children younger than 8 years were excluded because a comprehensive examination as well as a surgical correction of FT irregularities is quite rare in this age group [1]. In patients, an additional inclusion criterium was unilateral or bilateral decreased FT, diagnosed clinically by an orthopedic surgeon and confirmed with torsional MRI. Patients have been referred based on subjective complaints (hip or knee pain) or irregularities in walking and/or running that were spotted in their familial or social circles (school, athletic activities). Reduced femoral torsion during clinical examination (Craig’s Test) was confirmed with torsional MRI of the lower extremities, or CT in cases where performing an MRI was not possible on the grounds of compliance/tolerance. Patients with radiological evidence of reduced femoral torsion were referred for a gait analysis. The exclusion criteria for all participants were as follows: (1) contraindications for intervention due to medical issues; (2) neurological disorders; (3) previous derotation osteotomies of the femur; (4) other medical conditions that affect the gait; and (5) non-compliance or inability to follow the test procedures. Controls were excluded when decreased FT was manually measured (cut off <5° femoral anteversion) and if the kinematic hip rotation was at least one standard deviation (SD, ±) outside the values of other TDC in external rotation during walking. In total, 26 participants were included, 15 patients (6 female; 9 male) and 11 controls (6 female; 5 male), who were matched for age and gender. Patients’ mean age was 12.6 ± 2.2 years (9–17 years), and their mean body mass index (BMI) was 23.7 ± 4.3 kg/m^2^ (14.7–28.6 kg/m^2^). Controls’ mean age was 12.7 ± 2.4 (8–16 years) years, and the mean BMI was 18.8 ± 2.5 kg/m^2^ (15.6–23.5 kg/m^2^).

### 2.2. Measuring System and Procedure

The gait analysis protocol includes video documentation and kinematic analysis of movement around the hip, knee, and ankle joints, precise documentation of foot motion with the Oxford foot model, and electromyographic monitoring of key muscle groups. The walking and running area in the laboratory is limited to eight meters, with a fixed starting point for all participants to ensure consistency. Markers have been placed by experienced healthcare professionals who have been consistently performing gait analysis in our center, so as to minimize measurement errors. Additionally, the virtual knee alignment device (vKAD) is utilized for more accurate knee calculations.

All participants performed the 3D motion analysis barefoot on level ground during walking (WALK) at a self-selected velocity over a distance of eight meters. For the intervention running (RUN), participants were instructed to engage in moderate-intensity running, characterized by a pace faster than jogging yet slower than sprinting, typically falling within the mid-range of running velocities observed during gait analysis [29]. RUN mimics common sports practices for children. The order of conditions WALK and RUN was allocated by block randomization. Kinematic data were collected via a 16-camera Vicon System (Vicon Inc., Oxford, UK) with a minimum of five valid trials per leg.

Furthermore, subjective feelings of pain as well as engagement in athletic activities were documented in the frame of history-taking, and a clinical examination of the lower extremities was performed.

### 2.3. Data Processing

To evaluate the deviations of 3D gait kinematics in patients with decreased FT, the results of the two conditions RUN to WALK were compared. The within-group comparison was calculated separately for the patients and controls (patients RUN compared to WALK; controls RUN compared to WALK). The results of the controls served as a reference.

To find out whether the kinematic gait pattern differs between patients and controls, firstly, the between-groups comparison for both conditions, RUN and WALK, was calculated separately (patients WALK compared to controls WALK; patients RUN compared to controls RUN). Secondly, the differences (Δ) RUN–WALK for both of the groups were calculated separately (patients RUN–WALK, controls RUN–WALK) and compared between each other (Δ patients compared to Δ controls) to find out which of the kinematic gait differences of RUN–WALK were significant. All kinematic parameters were calculated during the stance phase (SP).

The passive hip range of motion (ROM), femoral and tibia torsion, and the difference mean velocity RUN–WALK were used to investigate their influence on the difference mean hip rotation of RUN–WALK in patients.

### 2.4. Statistical Analysis

All outcomes were described with the use of descriptive statistics, including means and the standard deviation (SD). The data were checked for normal distribution with the Shapiro–Wilk test. Patients and controls were matched for age and gender to exclude possible differences between the groups. This was tested with the T-test for independent samples. The mixed linear model was chosen, considering the dependence of both legs on the same person. It was used to compare the two conditions (RUN/WALK) and the two groups (patients/controls). The linear regression model was used to investigate the influence of the clinical examination and temporo-spatial parameter on the difference in mean hip rotation of RUN–WALK in patients. The significance level was set at *p* < 0.05.

This research was approved by the regional healthcare administration.

## 3. Results

Patients’ mean FT based on the clinical examination was −2.69 ± 8.27°; the controls’ mean FT was 15.45 ± 5.33° (*p* < 0.001). All patients, except one, were affected bilaterally. Patients and controls were matched for age (*p* = 0.890) and gender (*p* = 0.482). The BMI was significantly different in both groups (*p* = 0.001). A total of 66.7% of the patients experienced pain (*n* = 10) (once in the pelvis, twice in the hip, four times in the knee, and five times in the foot), and 33.3% of the patients experienced no pain (*n* = 5). None of the controls declared pain. The pain differed in both groups significantly (*p* < 0.001). A total of 77.3% of the patients (*n* = 11) and 81.8% of the controls (*n* = 9) practiced sports, which did not differ significantly between both groups (*p* = 0.622). Patients ran 3.36 m/s and walked 1.36 m/s; the controls ran 3.68 m/s and walked 1.40 m/s. The velocities did not differ significantly between both groups (running *p* = 0.059 and walking *p* = 0.531).

One child from the control group was excluded after the analysis due to a reduced femoral torsion in the manual clinical examination and alterations in the hip kinematics by more than one SD.

### 3.1. Kinematic Analysis during Walking and Running

**Figure 1:**
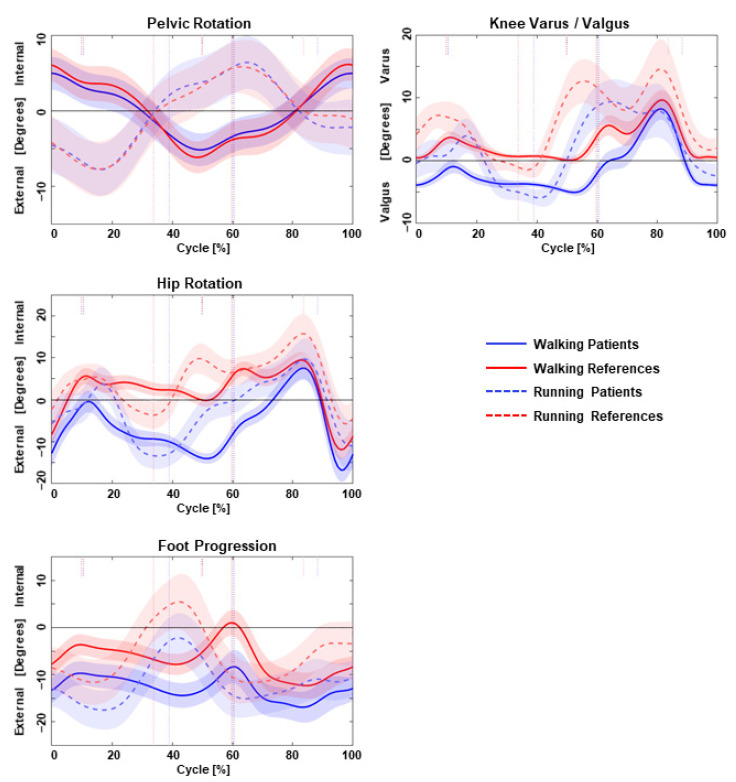
Kinematics between walking (solid line) and running (dashed line) for patients (blue) and references (red): transversal plane in the left column and frontal plane in the right column. Curves are plotted as mean values with a standard deviation.

The kinematic parameters of the pelvis, hip, knee, and ankle joints of the patient and controls during running (RUN) and walking (WALK) are presented in Table 1. An example of the kinematic traces is displayed in Figure 1.

Only the peak foot dorsiflexion (DF) showed a significant change (*p* < 0.001) during RUN compared to WALK in patients, which increased by 10.34°. The peak FPA increased by 4.09° in the direction out-toeing, which was not significant (*p* = 0.145). The same was seen in controls, with an increased peak foot DF of 11.85° (<0.001) and an increased peak FPA of 4.47° (*p* = 0.034) (Table 1).

During WALK, patients showed a significantly 9.30° higher peak hip external rotation (*p* = 0.004), a 5.28° higher peak knee valgus (*p* = 0.001), and a 5.19° higher peak FPA in the direction out-toeing (*p* = 0.037) compared to controls during SP (Table 1). Furthermore, patients showed an external mean hip rotation during WALK, while controls showed an internal mean hip rotation (*p* ≤ 0.001), with a difference of 11.47° (Table 1). During RUN, patients had a significant 5.12° higher peak knee valgus compared to controls (Table 1). Patients showed an external mean hip rotation during RUN, while controls showed an internal mean hip rotation with a difference of 7.65° (*p* = 0.539) (Table 1). Furthermore, patients showed a 10.41° higher peak hip external rotation, a 4.46° lower peak hip extension, and a 4.81° higher peak FPA in the direction of out-toeing compared to controls during RUN during SP, which were not statistically significant. The mean pelvic rotation in patients demonstrated external rotation during both walking (−0.06°) and running (−0.38°). In contrast, controls exhibited internal rotation of the pelvis during walking (−0.07°) and external rotation during running (−0.53°) (*p* = 0.008 and <0.001, respectively, for patients and controls). Although pelvic rotation per se showed significant variation when compared between running and walking among both patients and controls, no statistical significance was observed in the comparison of these values between patients and controls. The ROM exhibited during pelvic rotation was increased during running in the direction of internal rotation by approximately 5° among patients (*p* = 0.150) and similarly increased by roughly 3.7° when comparing walking and running among controls (*p* = 0.103), with no statistical significance achieved among movement status and participants’ groups.

### 3.2. Comparison of Running–Walking Differences between Patients and Controls

**Figure 2:**
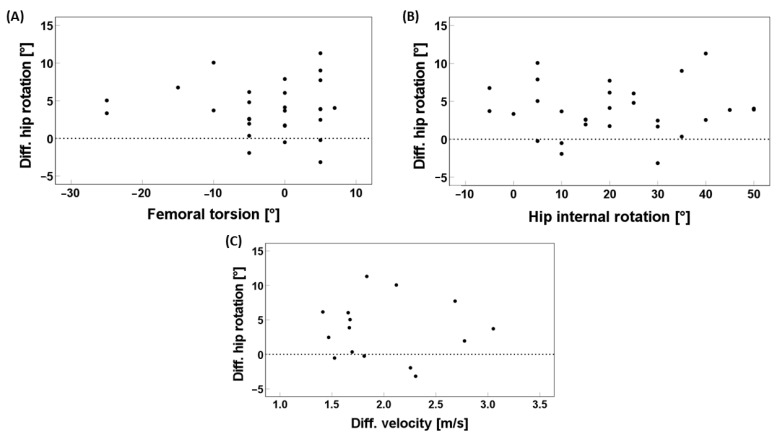
Linear regression of patients data; effect of femoral torsion measured in clinical examination (**A**), hip internal rotation measured by gait analysis (**B**) and difference mean velocity RUN-WALK measured by gait analysis (**C**) on the difference mean hip rotation RUN-WALK in patients with decreased FT; FT = femoral torsion; RUN = running; WALK = walking; Diff. = difference; (°) = degrees; and (m/s) = meter per second.

The differences from RUN–WALK compared between the two groups are presented in Table 2. In this regard, there was only a statistically significant difference for the mean hip rotation RUN–WALK. Patients turned their hip 3.82° more internally during RUN and stayed in an external hip rotation, whereas controls had the same rotation between WALK and RUN and stayed in an internal hip rotation (Table 2). No statistically significant variations in the differences from RUN-WALK were observed in the kinematics of the pelvis, the knee, and the ankle joint. The tibial torsion had a significant influence on the difference in mean hip rotation RUN–WALK (*p* = 0.024) with a Regression Coefficient B of −0.258 (Table 3). The other variables, namely the total hip rotation (*p* = 0.832), the hip external rotation (*p* = 0.939), the hip internal rotation (*p* = 0.939), the hip extension (*p* = 0.415), and the femoral torsion (*p* = 0.789), as well as the difference in mean velocity between RUN-WALK (*p* = 0.914), showed no effect on the difference in mean hip rotation RUN–WALK (Table 3 and Figure 2).

## 4. Discussion

This explorative study evaluated gait parameters measured during the stance phase (SP) of the gait cycle. Firstly, this study aimed to investigate deviations in 3D gait kinematics during running compared to walking in children with decreased FT. In patients, the gait deviations regarding DF and out-toeing increased during running compared to walking. Secondly, the comparison of the kinematic gait pattern between patients compared to TDC showed several kinematic differences for the gait pattern during walking. However, the kinematic differences for running achieved only statistical significance for the knee valgus. Importantly, the analysis of the difference mean hip rotation from running to walking showed a statistically significant difference in hip rotation in patients compared to TDC. Thirdly, for patients, this study investigated the influence of the findings of the clinical examination (Table 3) on the difference in mean hip rotation, measured from running to walking. Only the tibia torsion had a statistically significant effect on the difference in mean hip rotation.

The comparison of walking and running between patients and controls demonstrated increased foot DF and increased FPA in the out-toeing direction. The latter was more common among patients when running compared to walking. Dicharry, Dugan, and Bhat [30,31] report and interpret similar deviations in TD adults. Due to the increased vertical position of the tibia in running, more DF of the ankle is required to achieve initial contact and increases as the limb is loaded during running [30]. Due to shock absorption during SP in running, full foot pronation occurs, which may contribute to a forefoot abduction and explain the out-toeing foot [30,31]. In the other kinematic parameters, no notable deviations were observed. This corresponds with Franz et al. and Dicharry, who also found no change in the hip range of motion from walking to running and also found similar knee patterns in walking and running [30,32].

Furthermore, the present study supports and expands upon previously reported findings that these kinematic patterns are typical both for TD adults and children. These patterns are therefore not clinically relevant, and the same gait pattern is now demonstrated to also be found in children. In this frame, a tendency towards external rotation of the pelvis during running was shown. External pelvic rotation during running has also been underscored by Perpina-Martinez et al. (2023) in a kinematic analysis of 101 male and female adults [33]. Our study confirmed this tendency among children. Interestingly, the increase in the mean external rotation of the pelvis among adult males and females seems to be 2–4° higher than the respective increase among children with and without FR. Although a head-to-head comparison is not possible due to the different methods of gait analysis, it is likely that pelvic external rotation among running children is lower than among running adults.

Secondarily, the differences in the gait pattern between patients and TDC for walking, running, and the differences from running to walking were compared. For walking, the kinematic gait pattern differed in patients compared to controls. Patients in our study walked with more externally rotated hips compared to internally rotated hips in controls and showed more peak external rotation compared to controls during walking. This shows that our patients were not able to counterbalance their reduced FT due to their clinically reduced internal hip rotation, and thus more external mean hip rotation and more peak hip external rotation were measured in patients compared to controls during walking. Simultaneously, the mean of the pelvic rotation shifted slightly externally between walking and running among both patients and controls. Although this finding was only significant while comparing the walking to the running configuration, it can be assumed that greater pelvic exertion could partially compensate for hip external rotation during running among patients. Hence, the slight increase in external rotation of the pelvis and the decrease in external rotation of the hip might be mutually dependent.

Interestingly, Bruderer-Hofstetter et al. and Alexander et al. observed the opposite in patients with increased FT, where they walked with more internally rotated hips and had smaller external hip rotation compared to controls [26,27,34]. The higher knee valgus during walking in the kinematics of patients may result because patients had a higher BMI than controls, causing the knee to move more medially. Shultz et al. showed more genu valgum in children with a higher BMI. The increased FPA in the direction out-toeing, observed in patients during walking has been described by several authors [17,18]. Bruderer-Hofstetter et al. found the opposite FPA in patients with increased FT, whereby patients walked with decreased FPA compared to controls. The presented results suggest that the reduced FT of the hip is translated into increased external hip rotation of the entire leg and a more externally rotated foot position, and it seems that patients with decreased FT show the opposite gait pattern as patients with increased FT [26]. On the other hand, Lerch et al. found a moderate correlation between FT and FPA, where only one-fifth of the patients with decreased FT showed an out-toeing gate [6]. It is important to know that an out-toeing gate alone cannot be relied on as a diagnostic criterium for decreased.

Furthermore, the difference in mean hip rotation and peak hip external rotation were correlated with the presence of reduced femoral torsion (patients) in comparison to controls. Patients exhibited marked values of external hip rotation in comparison to controls. This is in line with the widely accepted concept of bone deformities having a dire impact on hip kinematics. However, according to a literature review of Bailly and colleagues, femoral torsion is poorly correlated with hip rotation among ambulant children with cerebral palsy [35]. Given that the study of Bailly does not analyze reduced femoral torsion per se, it is likely that its conclusions concern, in most cases, patients with increased femoral torsion. The kinematic profile of this bone deformity can be rather consistent with the gait pattern of cerebral palsy, which includes toeing and pelvic instability [4]. Additionally, it should be noted that the results of our analysis should be considered valid among children with no concomitant neurological disorders.

During running, the same kinematic gait pattern was observed as during walking, compared between the two groups, but was only significant for the knee valgus. Most deviations were probably not significant due to the smaller groups. It is clinically relevant to observe that patients were still not able to counterbalance their reduced FT during running and showed the same tendencies as during walking. In the long term, this indicates that the knee joint tends to receive biomechanically adverse loading in both walking and running gait, potentially sustaining constant microdamage during the most prominent gait patterns. While the long-term risks of these loading conditions are yet to be appraised, evidence suggests that prolonged knee valgus stress can lead to patellofemoral pain syndrome and anterior cruciate ligament (ACL) injury [36]. Running to walking, compared between the two groups, patients turned their hips 3.82° more internally during running and stayed in an external mean hip rotation, whereas controls kept the same internal mean hip rotation. Patients may show this deviation to extend the foot lever so they can push off more easily or to avoid pain that can possibly be caused by compression in the joint or FAI. This study showed that 66.7% of the patients experienced pain, and several authors agree that decreased FT can lead to hip and knee pain that may result in difficulties in daily activities and sports [17,19,21].

For the third aim, the tibia torsion showed an influence on the difference in mean hip rotation from running to walking in patients, which was only −0.258°. This small effect can be interpreted as a measurement error and is unlikely to hold clinical relevance. Therefore, neither the passive ROM of the hip, torsions, nor the difference in mean velocity RUN-WALK had an effect on the difference in mean hip rotation RUN-WALK.

### Limitations and Future Research

This study is subject to a number of limitations regarding the gait analysis protocol and capacity. The walking and running area in the laboratory was limited to a distance of eight meters. To mitigate the effect of this on the study outcomes, the starting point was a fixed line on the floor, which was the same for all participants. Adequate space to accelerate and decelerate was available at both the starting and ending points to ensure that motion initiation and cessation are not abrupt and do not put study participants at risk of injury. Secondly, controls FT was not verified in MRI compared to patients. Although, the accuracy of the clinical examination of the torsional profile is quite high [37], we cannot exclude that some controls might have had decreased FT. To minimize this possibility, apart from the clinical measurement of FT, controls whose kinematic hip rotation was one SD outside the values of other TD’s in external rotation were deemed to be excluded. Finally, gait analysis per se has inherent measurement limitations [38]. Hip rotation kinematics have been reported as the least repeatable parameter in clinical 3D gait analysis, with imprecise marker placement being the main source of error [38]. To minimize these errors, measurements were performed in a strict, standardized way by experienced healthcare personnel. Both a static and dynamic trial were performed to identify imprecise marker placement, which was replaced when necessary [39]. Additionally, in the standardized marker model, the virtual knee alignment device (vKAD) was used for more precise knee calculations. The control group was slightly smaller than the study group given the availability of eligible patients and control subjects who consented to participate in the study during the recruiting period. Moreover, the coexistence of frontal/coronal deformities of the lower extremities (e.g., genua valga or genua vara) was not taken into account in the analysis.

Further research is needed to obtain a more extensive overview of all possible 3D kinematic gait deviations and should also include kinetics as well as EMG measurements to provide information on biomechanical aspects. Additionally, incorporating evidence-based and population-specific measurements of quality of life needs to be prioritized in future research. The somatometric documentation of the participants in this study revealed a noteworthy trend in this regard; patients exhibited a higher BMI in comparison to controls. Although BMI is associated with multiple genetic and lifestyle factors, further investigation could shed light on the potentially limiting impact of reduced FT on physical activity and the consequent tendency towards increased BMI. Although children younger than 8 years were not included due to the rarity of derotation osteotomies of the femur in this demographic [1], collecting and analyzing data from this age group could enhance our understanding of physiological musculoskeletal development. It could also potentially improve the design of orthopedic orthotics for the hip within this age group by informing manufacturers and prescribers about relevant motion patterns. Overall, delivering a comprehensive functional assessment by means of both functional gait measurements and documentation of subjective perceptions has a major potential to support personalized decisions for the management of decreased FT.

## 5. Conclusions

Overall, there are gait deviations within and between patients with decreased FT and TDC during walking and running. Increased foot dorsiflexion, forefoot progression angle in the direction of out-toeing, and knee valgus stress were observed during walking and running among children with decreased femoral torsion (FT). These kinematic patterns are in line with those observed in adults. Moreover, a subtle tendency towards external rotation of the pelvis and a persistent external hip rotation during running are noted. These findings, which, to the best of our knowledge, have not been reported among children so far, underscore the potential of gait analysis to improve the evaluation of children with decreased FT and guide personalized decision-making. They namely enrich clinicians’ understanding of decreased FT as an anatomical configuration with implications for walking and running, two predominant activities in the lifestyle of most underage individuals. Clinicians could harness this objective measure of limitations their patients experience in daily living settings to improve their decision making. This confers benefits to patients and their families, so children and adolescents can face challenges by articulating the limitations that they face while walking and running. Gait analysis helps physician to comprehend their complaints and provides a common functional basis for setting functional goals and reaching a shared decision rather than adhering to a treatment plan articulated in terms they might find difficult to understand.

## Figures and Tables

**Table 1:** Kinematics during walking and running in patients with decreased femoral torsion and controls.

Groups	Patients		Controls		Patients	Controls	Patients/Controls	Patients/Controls
Conditions Parameters	WALK (Mean ± SD)	RUN (Mean ± SD)	WALK (Mean ± SD)	RUN (Mean ± SD)	RUN/WALK (*p* Value)	RUN/WALK (*p* Value)	WALK (*p* Value)	RUN (*p* Value)
Mean pelvic rotation (°) Internal(+)/external rotation(−)	−0.06 ± 2.72	−0.38 ± 2.63	0.07 ± 1.05	−0.53 ± 2.04	0.008 *	<0.001 *	0.474	0.216
ROM pelvic rotation (°) Internal(+)/external rotation(−)	12.38 ± 3.96	17.49 ± 4.04	14.33 ± 3.86	17.99 ± 5.54	0.150	0.103	0.231	0.529
Mean hip rotation (°) Internal(+)/external rotation(−)	−8.80 ± 8.08	−4.96 ± 9.00	2.67 ± 5.92	2.69 ± 8.21	0.680	0.936	<0.001 *	0.539
Peak hip external rotation (°) Internal(+)/external rotation(−)	−17.08 ± 7.80	−17.16 ± 8.84	−7.78 ± 6.40	−6.75 ± 7.46	0.999	0.777	0.004 *	0.503
Peak hip extension (°) Flexion(+)/extension(−)	−6.64 ± 5.65	−4.56 ± 6.75	−10.45 ± 6.29	−9.02 ± 6.82	0.310	0.707	0.071	0.292
Peak knee valgus (°) Varus(+)/Valgus(−)	−6.55 ± 3.24	−7.07 ± 3.73	−1.27 ± 1.93	−1.95 ± 3.81	0.741	0.543	0.001 *	0.034 *
Peak foot DF (°) Dorsi(+)/plantarflexion(−)	13.23 ± 3.02	23.57 ± 3.94	14.78 ± 3.04	26.63 ± 3.93	<0.001 *	<0.001 *	0.179	0.052
Peak foot PA (°) In(+)/out-toeing(−)	−11.31 ± 9.08	−15.40 ± 5.95	−6.12 ± 4.65	−10.59 ± 6.60	0.145	0.034 *	0.037 *	0.107

Mean ± SD of kinematic parameters in patients with decreased FT and controls in all conditions (patients RUN, patients WALK, controls RUN, controls WALK), the comparison within-groups (patients RUN/WALK, controls RUN/WALK) and between-groups (patients/controls WALK, patients/controls RUN) for both conditions, during stance phase (SP); FT = femoral torsion, WALK = walking, RUN = running, DF = dorsiflexion, PA = progression angle, (°) = degrees, and SD = standard deviation. * Significant difference: *p* < 0.05.

**Table 2:** Kinematics of the differences from running to walking in patients with decreased femoral torsion and controls.

Groups Conditions Parameters	Patients RUN-WALK (Mean ± SD)	Controls RUN-WALK (Mean ± SD)	Δ Patients/Δ Controls (*p* Value)
Δ mean pelvic rotation (°) Internal(+)/external rotation(−)	−0.32 ± 1.88	−0.61 ± 1.61	0.179
Δ ROM pelvic rotation (°) Internal(+)/external rotation(−)	5.11 ± 5.84	3.67 ± 4.67	0.601
Δ mean hip rotation (°) Internal(+)/external(−)rotation	3.84 ± 3.40	0.02 ± 4.30	0.005 *
Δ peak hip external rotation (°) Internal(+)/external(−)rotation	−0.09 ± 3.17	1.03 ± 5.12	0.490
Δ peak hip extension (°) Flexion(+)/extension(−)	2.08 ± 4.74	1.43 ± 3.38	0.778
Δ peak knee valgus (°) Varus(+)/Valgus(−)	−0.52 ± 1.77	−0.67 ± 2.88	0.944
Δ peak foot DF (°) Dorsi(+)/plantarflexion(−)	10.34 ± 4.30	11.85 ± 4.34	0.357
Δ peak foot PA (°) In(+)/out(−)-toeing	−4.08 ± 6.64	−4.47 ± 4.19	0.902
Δ mean velocity (m/s)	2.00 ± 0.51	2.28 ± 0.35	0.104

Mean ± SD of kinematic parameters and temporo-spatial parameter in patients with decreased FT and controls for the differences from running to walking (patients RUN-WALK, controls RUN-WALK) and between-groups comparison (Δ patients/Δ controls) during stance phase (SP); FT = femoral torsion, RUN = running, WALK = walking, DF = dorsiflexion, PA = progression angle, Δ = difference (°) = degrees, (m/s) = meter per second, SD = standard deviation. * Significant difference: *p* < 0.05.

**Table 3:** Influence of the clinical examination and temporo-spatial variable on the difference mean hip rotation running to walking in patients with decreased femoral torsion. *p*-values rendering statistical significance have been highlighted in bold and marked with an asterisque (*).

Variables	Regression Coefficient B	*p* Value	Patients (Mean ± SD)	Controls (Mean ± SD)
Total hip rotation (°)	0.009	0.832	89.83 ± 16.39	91.82 ± 16.80
Hip external rotation (°)	0.003	0.939	69.14 ± 14.34	43.64 ± 14.57
Hip internal rotation (°)	0.003	0.939	20.69 ± 15.74	48.18 ± 10.86
Hip extension (°)	0.124	0.415	11.90 ± 4.31	14.32 ± 4.70
Femoral torsion (°)	−0.021	0.789	−2.69 ± 8.27	15.45 ± 5.33
Tibia torsion (°)	−0.258	0.024 *	−23.45 ± 5.53	−20.55 ± 6.68
Δ mean velocity RUN-WALK (m/s)	−0.254	0.914	2.00 ± 0.51	2.28 ± 0.35

## Data Availability

The data presented in this study are available on request from the corresponding author. The data are not publicly available due to restrictions, e.g., privacy or ethical.
